# The Type IX Secretion System (T9SS): Highlights and Recent Insights into Its Structure and Function

**DOI:** 10.3389/fcimb.2017.00215

**Published:** 2017-05-26

**Authors:** Anna M. Lasica, Miroslaw Ksiazek, Mariusz Madej, Jan Potempa

**Affiliations:** ^1^Department of Oral Immunology and Infectious Diseases, University of Louisville School of DentistryLouisville, KY, United States; ^2^Department of Bacterial Genetics, Faculty of Biology, Institute of Microbiology, University of WarsawWarsaw, Poland; ^3^Department of Microbiology, Faculty of Biochemistry, Biophysics, and Biotechnology, Jagiellonian UniversityKrakow, Poland

**Keywords:** secretion, T9SS, *Porphyromonas gingivalis*, pathogenesis, gliding motility, proteins, virulence

## Abstract

Protein secretion systems are vital for prokaryotic life, as they enable bacteria to acquire nutrients, communicate with other species, defend against biological and chemical agents, and facilitate disease through the delivery of virulence factors. In this review, we will focus on the recently discovered type IX secretion system (T9SS), a complex translocon found only in some species of the *Bacteroidetes* phylum. T9SS plays two roles, depending on the lifestyle of the bacteria. It provides either a means of movement (called gliding motility) for peace-loving environmental bacteria or a weapon for pathogens. The best-studied members of these two groups are *Flavobacterium johnsoniae*, a commensal microorganism often found in water and soil, and *Porphyromonas gingivalis*, a human oral pathogen that is a major causative agent of periodontitis. In *P. gingivalis* and some other periodontopathogens, T9SS translocates proteins, especially virulence factors, across the outer membrane (OM). Proteins destined for secretion bear a conserved C-terminal domain (CTD) that directs the cargo to the OM translocon. At least 18 proteins are involved in this still enigmatic process, with some engaged in the post-translational modification of T9SS cargo proteins. Upon translocation across the OM, the CTD is removed by a protease with sortase-like activity and an anionic LPS is attached to the newly formed C-terminus. As a result, a cargo protein could be secreted into the extracellular milieu or covalently attached to the bacterial surface. T9SS is regulated by a two-component system; however, the precise environmental signal that triggers it has not been identified. Exploring unknown systems contributing to bacterial virulence is exciting, as it may eventually lead to new therapeutic strategies. During the past decade, the major components of T9SS were identified, as well as hints suggesting the possible mechanism of action. In addition, the list of characterized cargo proteins is constantly growing. The actual structure of the translocon, situated in the OM of bacteria, remains the least explored area; however, new technical approaches and increasing scientific attention have resulted in a growing body of data. Therefore, we present a compact up-to-date review of this topic.

## Introduction

Secretion of hemolysin A by *E. coli*, described four decades ago, was the first protein secretion system discovered in Gram-negative bacteria (diderm bacteria; Goebel and Hedgpeth, 1982). Since then, eight other protein secretion pathways have been characterized in these prokaryotes, which have a cell envelope consisting of the inner membrane (IM) and the outer membrane (OM) separated by the periplasm. They are now referred to as type *x* secretion systems (T1SS–T9SS; reviewed in Abdallah et al., 2007; Gerlach and Hensel, 2007; Remaut et al., 2008; Desvaux et al., 2009; Goyal et al., 2014; Costa et al., 2015; Abby et al., 2016). Secretion systems in diderm bacteria are considered gateways through the OM that transport cargo with the help of either dedicated IM and periplasmic proteins or the Sec, Tat, and holins systems that first transport cargo to the periplasm. In fact, the Sec, Tat, and holins pathways, which transport proteins across the cytoplasmic membrane, are universal among bacteria, eukaryotes, and even archaea (Hutcheon and Bolhuis, 2003; Denks et al., 2014; Berks, 2015; Saier and Reddy, 2015). Therefore, secretion may be either a single-step process in which substrates (proteins or DNA) are translocated through a designated cell envelope-spanning structure (T1SS, T3SS, T4SS, and T6SS) or a two-step process in which the substrates first cross the IM into the periplasm using the Sec/Tat/holins systems, then are directed to the OM translocon. The final destinations of secreted cargos are diverse: they may stay attached to the surface of the OM, be released into the extracellular milieu, or be injected into the cytoplasm of a target cell (Costa et al., 2015; Abby et al., 2016).

Secretion systems perform numerous physiological functions essential for cell propagation and fitness within a specific ecological niche. They facilitate nutrient acquisition, communication with the environment, attachment to various surfaces, defense against host antimicrobial systems, and delivery of virulence factors at a precise location such as a eukaryotic cell (Letoffe et al., 1994; Henke and Bassler, 2004; Gerlach and Hensel, 2007; Rondelet and Condemine, 2013; Gaytan et al., 2016; Hachani et al., 2016; Majerczyk et al., 2016). However, none of the above adaptations can be assigned solely to one type of secretion.

The presence of protein secretion systems varies among phylogenetic lineages of diderm bacteria. *Proteobacteria* encode the broadest range of described secretion types, whereas other clades have a strong preference for only one or two types (e.g., *Fusobacteria* possess only T5SS; *Chlamydiae*, T3SS and T5SS). The most widespread systems are T1SS and T5SS; conversely, T2SS is rarely detected outside *Proteobacteria* (Abby et al., 2016).

In this review, we will cover the current knowledge regarding the recently discovered type IX secretion system (T9SS), also known as the Por secretion system (PorSS) or PerioGate. T9SS is exclusively present in the *Bacteroidetes* phylum, in a majority of its species (62% out of 97 genomes available; Sato et al., 2010; McBride and Zhu, 2013; Abby et al., 2016).

## Discovery of T9SS

Uncovering and characterizing this unique secretion system was a gradual process over the last two decades and originated from studies of the Gram-negative, non-motile, anaerobic bacterium *Porphyromonas gingivalis*. *P. gingivalis* is a human oral pathogen that is a major causative agent of periodontitis, and, along with two other bacteria, *Tannerella forsythia* and *Treponema denticola*, forms the so-called red complex (Hajishengallis, 2015). Besides being a key pathogen in periodontitis, *P. gingivalis* is implicated in many systemic illnesses such as atherosclerosis (Kebschull et al., 2010), aspiration pneumonia (Benedyk et al., 2016), rheumatoid arthritis (RA; Laugisch et al., 2016), and even cancer (Whitmore and Lamont, 2014; Gao et al., 2016).

An important initial finding was that *P. gingivalis* produces potent proteolytic enzymes called gingipains (Kgp, RgpA, and RgpB; discussed in more detail later in this review; Pike et al., 1994; Pavloff et al., 1995; Curtis et al., 1999). Gingipains are essential virulence factors responsible for corrupting host innate defense mechanisms (Potempa et al., 2003; Hajishengallis, 2015). They are secreted in large amounts and are mainly attached to the surface of the OM, but are also partially released in a soluble form into the extracellular milieu (Pike et al., 1994; Rangarajan et al., 1997). Because none of the genes associated with known protein secretion systems could be found in the *P. gingivalis* genome, it was suspected that this bacterium had developed a unique OM translocon.

**Figure 1:**
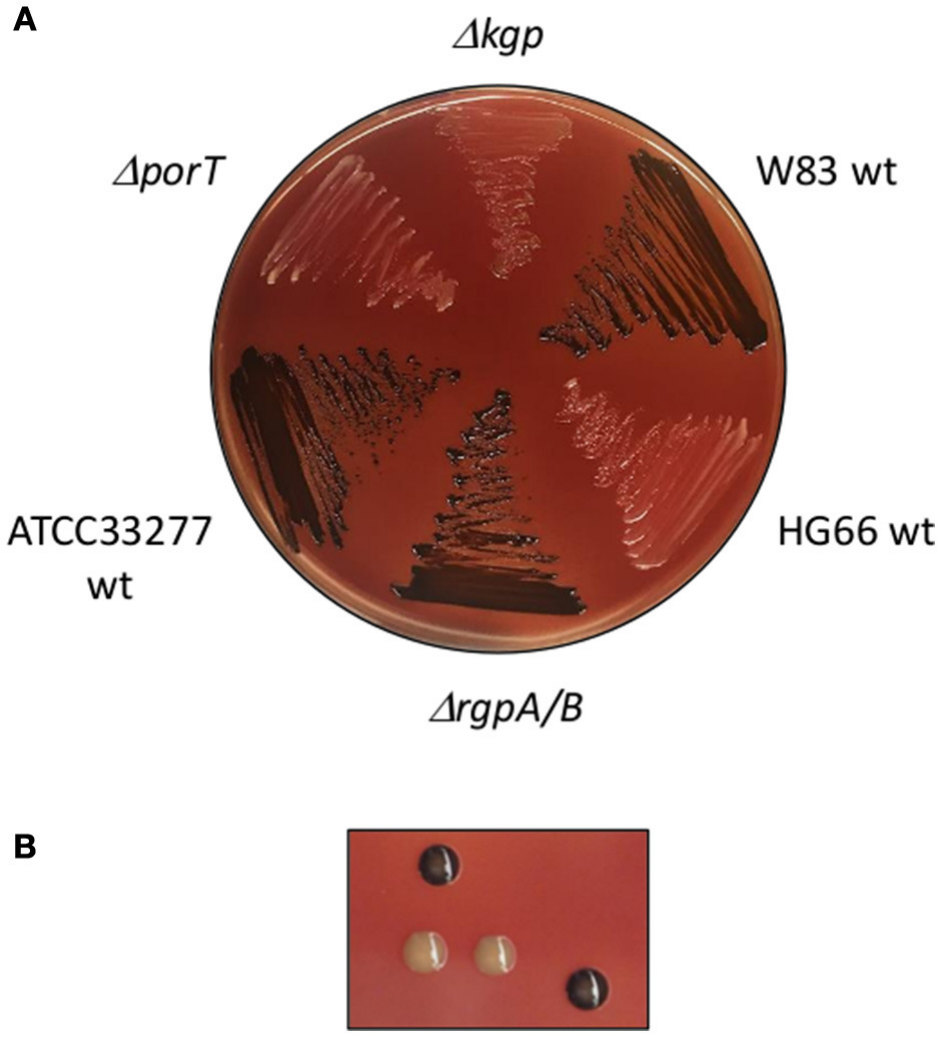
**Pigmentation of various ***P. gingivalis*** W83 strains. (A)** The wild-type *P. gingivalis* W83 and ATCC33277 strains grown anaerobically on blood agar plates present brown/black pigmentation due to heme accumulation. This phenotype is in a great part dependent on Kgp gingipain activity on the cell surface. *P. gingivalis* strains deficient in Kgp activity yield beige colonies which darken over the time. Arginine gingipains (RgpA/B) are not involved in this process and their deletion does not influence pigmentation. Strains impaired in T9SS e.g., Δ*porT* lack pigmentation which is never restored. Due to the absence of A-LPS in the *P. gingivalis* HG66 strain all gingipains and other T9SS cargo proteins are not associated with the cell membrane, but secreted into extracellular milieu resulting in white phenotype. **(B)** Single colonies of *P. gingivalis* strains grown for 7 days showing black or white pigmentation.

The search for this novel secretion system was greatly facilitated by the observation that colonies of *P. gingivalis* deficient in gingipain activity lack black pigmentation while growing on blood agar plates (Figure 1; Okamoto et al., 1998; Shi et al., 1999). Colony pigmentation results from the accumulation of heme on the surface of *P. gingivalis* cells, a process dependent on the proteolytic activity and hemagglutinin- and heme/hemoglobin-binding activity of gingipains (Smalley et al., 1998; Sroka et al., 2001). Spontaneous white/beige mutants were occasionally observed, and this phenotype was associated with, among other things, decreased cell surface-associated proteolytic activity (McKee et al., 1988; Shah et al., 1989). The discovery of the essential role of secreted, cell-bound gingipains in heme acquisition meant that pigmentation could be used as an easy screening tool for mutations blocking gingipain secretion. Of note, as potent virulence factors, gingipains were of particular interest for elucidating the role of *P. gingivalis* in the development of periodontitis.

Several high-throughput transposon mutagenesis studies were performed, resulting in the characterization of various pigment-less clones. Early studies associated this phenotype with the impaired activity of trypsin-like proteases and diminished hemagglutination and heme acquisition by mutants (Hoover and Yoshimura, 1994; Genco et al., 1995). Later investigations found aberrations in polysaccharide synthesis and disruption of *kgp* (one of the gingipains; Simpson et al., 1999; Chen et al., 2000; Abaibou et al., 2001; Shoji et al., 2002). Finally, Sato et al. (2005) identified in their transposon study *porT* (PG0751/PGN_0778), the first gene encoding a protein involved in the secretion of gingipains. Their mutated, non-pigmented strain had impaired gingipain activity. Moreover, gingipains accumulated in the periplasm as enzymatically inactive proenzymes instead of being exported outside the cell. A database search (BLASTP) found that PorT is present only in some species of the *Bacteroidetes* phylum, such as *Porphyromonas gingivalis, Cytophaga hutchinsonii*, and *Prevotella intermedia*, and absent from many other phylum proteomes like *Bacteroides thetaiotaomicron* and *Bacteroides fragilis* (Sato et al., 2005). Two years later, another gene, *sov* (PG0809/PGN_0832), was implicated in the secretion of gingipains, showing a mutation phenotype identical to the one observed for the *porT* mutation (Saiki and Konishi, 2007).

Finally, the 2010 comparison of the *porT*-positive proteomes/genomes of *C. hutchinsonii* and *P. gingivalis* with the *porT*-negative species *B. thetaiotaomicron* resulted in a list of 55 genes (in addition to *porT*) potentially involved in the secretion mechanism. Subsequent isogenic mutagenesis of all selected genes resulted in the identification of 11 genes (including *porT* and *sov*) associated with gingipain transport across the OM and gingipain activation. Because these proteins do not have sequence similarity to components of any other known secretion system, it was assumed to be a novel secretion system and was originally called the Por secretion system (PorSS; Sato et al., 2010; Nakayama, 2015). To be consistent with the existing nomenclature of secretion systems in diderm bacteria, the system was later designated the type IX secretion system or T9SS.

## New secretion system: A deadly weapon or a peaceful tool?

The comparative analysis of genomes carried out in a search for *porT* homologs revealed that T9SS is exclusively present in the *Bacteroidetes* phylum (Sato et al., 2005). Numerous studies on *P. gingivalis* show that T9SS is involved in virulence factor secretion, which damages human tissues and dysregulates immune responses (Potempa et al., 2003; Yoshimura et al., 2008; Sato et al., 2013; Bielecka et al., 2014; Taguchi et al., 2015). In addition, *T. forsythia* and *Prevotella intermedia* (another oral pathogenic bacteria) use this secretion pathway to disseminate their effector proteins (Nguyen et al., 2007; Veith et al., 2013; Narita et al., 2014; Tomek et al., 2014; Ksiazek et al., 2015b). Consequently, it is plausible that more pathogens from the *Bacteroidetes* phylum carrying *porT* homologs are utilizing this mechanism for virulence factor secretion. Although no experimental data are available to support this, it is likely that T9SS is a molecular weapon aimed at various host cells, similar to many other secretion systems (especially T3SS and T6SS).

Among *Bacteroidetes' porT*-positive species, there are many non-pathogenic environmental microorganisms such as *C. hutchinsonii* and *F. johnsoniae*. Both bacteria are aerobes ubiquitously distributed in soil and are capable of digesting macromolecules such as cellulose and chitin, respectively (Stanier, 1942, 1947). They are motile microorganisms that use a movement mechanism called gliding motility (Jarrell and McBride, 2008; Nakane et al., 2013). Surprisingly, the core T9SS genes are a subset of those necessary for gliding (*gldK: ortholog of P. gingivalis porK, gldL/porL, gldM/porM, gldN/porN, sprA/sov, sprE/porW*, and *sprT/porT*; Sato et al., 2010; McBride and Zhu, 2013; Shrivastava et al., 2013; McBride and Nakane, 2015). Moreover, secretion of chitinase and cellulase requires T9SS, meaning the system functions as a non-invasive tool used for movement and food acquisition in these bacteria (Kharade and McBride, 2014; Zhu and McBride, 2014; Yang et al., 2016).

The detailed mechanisms and regulation of T9SS in gliding motility and food scavenging are still under investigation and may reveal additional functions (even in non-gliding species).

## Structural and functional components of *P. gingivalis* T9SS

Presently, 18 genes from a total of 29 candidates have been proven essential for proper T9SS function in *P. gingivalis* by deletion mutagenesis studies (Heath et al., 2016). Deletion of any of these genes results in the white pigmentation phenotype and accumulation of cargos (e.g., gingipains) in the periplasm. Some of these proteins build the core structures in the IM and OM, some play regulatory or accessory roles, and others are involved in post-translationally modifying cargo proteins (Table 1). Many aspects of their functions have yet to be discovered.

**Table 1:** **T9SS components**.

**Locus Tag**			***Porphyromonas gingivalis*** **W83**					
**W83 NC_002950.2**		**ATCC33277 NC_010729.1**	**Protein accession number**	**Protein description**	**Mol weight (kDa)^a^**	**Interactions ^b^*in vitro*^c^*in vivo***	**Homologs *T. forsythia* ATCC 43037–Tanf *F. johnsoniae* UW101-Fjoh**	**References^d^**
**CYTOPLASMIC AND INNER MEMBRANE COMPONENTS**								
PG_RS04080	PG0928	PGN_1019	WP_005875211.1	PorX; chemotaxis protein CheY, cytoplasmic protein	60.6	PorY^b^, SigP^b^, PorL^b^	Tanf_12330 Fjoh_2906	Sato et al., 2010; Kadowaki et al., 2016; Vincent et al., 2016
PG_RS00240	PG0052	PGN_2001	WP_005873974.1	PorY; sensor histidine kinase, inner membrane protein	44.6	PorX^b^	Tanf_13050 Fjoh_1592	
PG_RS01295	PG0289	PGN_1675	WP_012458450.1	PorL, inner membrane protein	34.8	PorM^b^^,^^c^	Tanf_02365 Fjoh_1854 (GldL)	Sato et al., 2010; Gorasia et al., 2016; Kadowaki et al., 2016; Vincent et al., 2016, 2017
PG_RS01300	PG0290	PGN_1674	WP_005874203.1	PorM, inner membrane protein	56.4	PorL/K/N^c^	Tanf_02370 Fjoh_1855 (GldM)	Sato et al., 2010; Gorasia et al., 2016; Kadowaki et al., 2016; Vincent et al., 2017
**PERIPLASMIC COMPONENTS**								
PG_RS01305	PG0291	PGN_1673	WP_005874243.1	PorN	41.3	PorP^b^,PorK/L/M^c^ PG0189^c^	Tanf_02375 Fjoh_1856 (GldN)	Sato et al., 2010; Gorasia et al., 2016; Kadowaki et al., 2016; Vincent et al., 2017
PG_RS01290	PG0288	PGN_1676	WP_043876477.1	PorK; lipoprotein	54.1	PorN^c^, PorM/P^b^PG0189^c^	Tanf_02360 Fjoh_1853 (GldK)	Sato et al., 2010; Gorasia et al., 2016; Kadowaki et al., 2016; Vincent et al., 2017
PG_RS08590	PG1947	PGN_1877	WP_005873869.1	PorW; lipoprotein	132.1	n.d.^e^	Tanf_00060 Fjoh_1051 (SprE)	Sato et al., 2010
PG_RS04660	PG1058	PGN_1296	WP_005873448.1	Lipoprotein; TPRd, WD40d, CRDd, OmpA Family domain	74.9	n.d.	Tanf_02260 Fjoh_1647^f^	Heath et al., 2016
**OUTER MEMBRANE AND SURFACE COMPONENTS**								
PG_RS03550	PG0809	PGN_0832	WP_012457811.1^g^	Sov; β-barrel protein	281.1	n.d.	Tanf_04410 Fjoh_1653 (SprA)	Saiki and Konishi, 2007, 2010b; Sato et al., 2010; Kadowaki et al., 2016
PG_RS02670	PG0602	PGN_0645	WP_010956079.1	PorQ; β-barrel protein	37.9	n.d.	Tanf_12465 Fjoh_2755	Sato et al., 2010
PG_RS01285	PG0287	PGN_1677	WP_005874180.1	PorP; β-barrel protein	35.0	PorN/K^b^	Tanf_02355 Fjoh_3477^h^	Sato et al., 2010; Kadowaki et al., 2016; Vincent et al., 2017
PG_RS03295	PG0751	PGN_0778	WP_039417575.1	PorT; β-barrel protein	26.7	n.d.	Tanf_10520 Fjoh_1466 (SprT)	Sato et al., 2005, 2010; Nguyen et al., 2007; Kadowaki et al., 2016
PG_RS00125	PG0027	PGN_0023	WP_004583425.1	PorV (LptO); β-barrel protein	43.1	PorU^c^	Tanf_04220 Fjoh_1555	Ishiguro et al., 2009; Sato et al., 2010; Chen et al., 2011; Glew et al., 2012; Saiki and Konishi, 2014
PG_RS00870	PG0189	PGN_0297	WP_005874727.1	β-barrel protein	25.6	PorK/N^c^	Tanf_09815 Fjoh_1692	Gorasia et al., 2016
PG_RS02385	PG0534	PGN_1437	WP_005875072.1	TonB-dependent receptor; β-barrel protein	92.3	n.d.	Tanf_07980 Fjoh_0118	Saiki and Konishi, 2010a
PG_RS00885	PG0192	PGN_300	WP_043876475.1	Omp17; OmpH-like	19.6	n.d.	Tanf_09800 Fjoh_1689^i^	Taguchi et al., 2015
PG_RS00120	PG0026	PGN_0022	WP_005874469.1	PorU; surface C-terminal signal peptidase	128.2	PorV (LptO)^c^	Tanf_02580 Fjoh_1556	Sato et al., 2010; Glew et al., 2012; Saiki and Konishi, 2014; Gorasia et al., 2015
PG_RS07070	PG1604	PGN_0509	WP_010956350.1	PorZ; surface B-propeller protein	83.6	n.d.	Tanf_12435 Fjoh_0707	Glew et al., 2014; Lasica et al., 2016

a *Calculated from amino acid sequence including a signal peptide*.

b *In vitro experiments*.

c *In vivo experiments*.

d *References to original papers pertinent only to P. gingivalis T9SS. Proteomic papers are not cited in the table but they are referred in the text*.

e *Not determined*.

f *F. johnsoniae possesses 5 proteins homologous to PG1058, the one with the highest score is given in the table. All 5 F. johnsoniae proteins (Fjoh_1647, Fjoh_4540, Fjoh_3950, Fjoh_3973, Fjoh_3476) range between 26–29% identities with 90-98% coverage comparing to PG1058 (assessed by NCBI BLAST)*.

g *Accession number given in the table is for the Sov protein from P. gingivalis ATCC33277 due to miss-annotation in W83 genome as two separate ORFs (PG0809/PG0810; Saiki and Konishi, 2007)*.

h *F. johnsoniae possesses numerous homologous proteins to PG0287 (PorP), the one with the highest score is given in the table. Five proteins with the highest overall score (Fjoh_3477, Fjoh_3951, Fjoh_1646, Fjoh_4539, Fjoh_2274) range between 25–29% identities with 86–93% coverage and gaps 3–9% comparing to PG0287 (PorP). The most explored PorP-like protein of F. johnsoniae is Fjoh_0978 (SprF; Rhodes et al., 2011) however, it has lower scores comparing to the proteins mentioned above (22% identities, 83% coverage, 18% gaps) as assessed by NCBI BLAST*.

i *The closest homolog in F. johnsoniae proteome is Fjoh_1689 is much larger protein than its equivalent in P. gingivalis (341 residues vs. 174; 32% identities, 95% PG0192 coverage assessed by NCBI BLAST)*.

**Figure 2:**
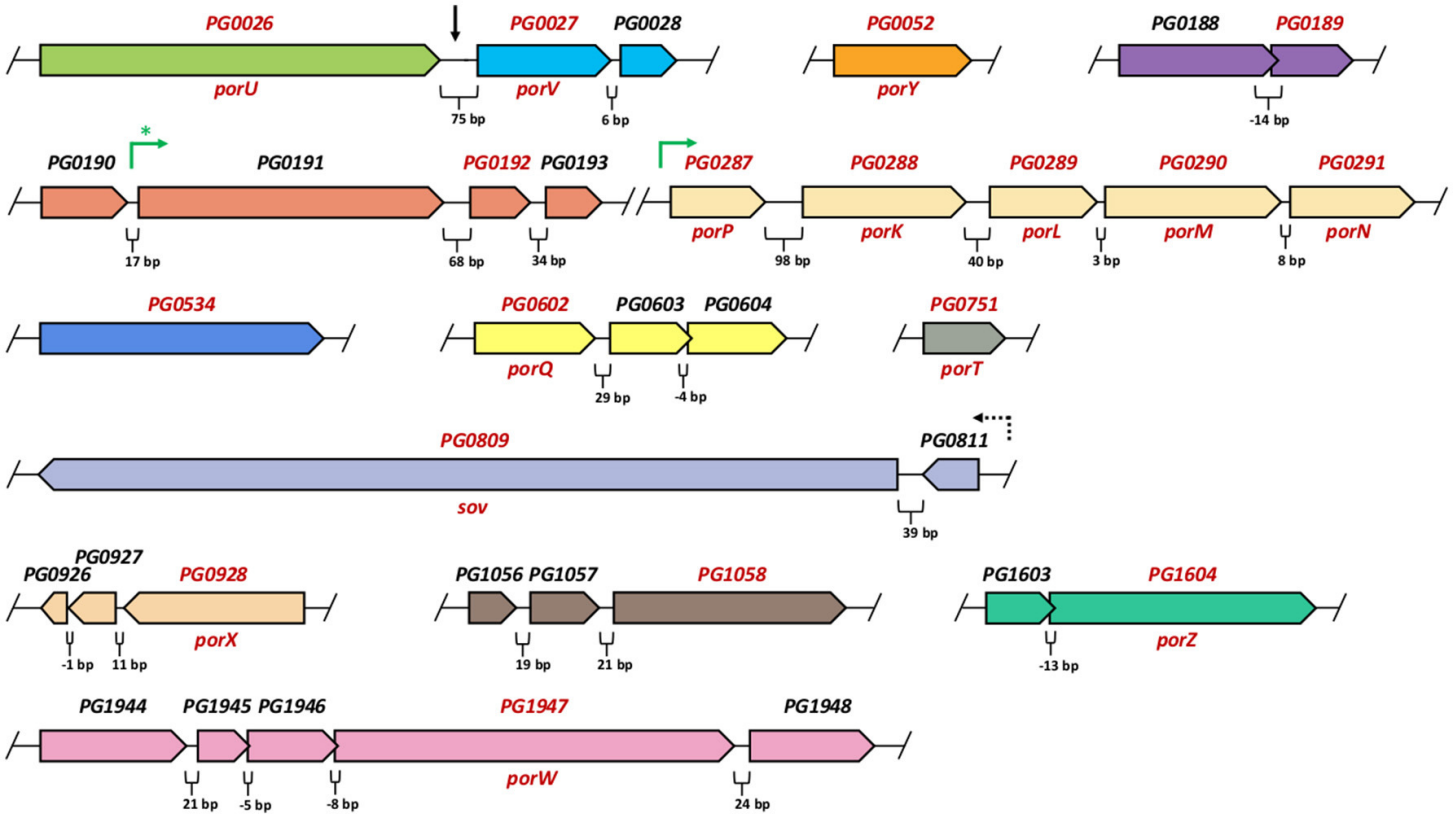
**Arrangement of ***P. gingivalis*** W83 genes encoding T9SS components**. Genes are grouped according to *in silico* operon predictions, reflecting direction of transcripts (Dam et al., 2007; Mao et al., 2009; Pertea et al., 2009; Taboada et al., 2012). Gaps in the genome are indicated by the slashes. Intervals between adjacent genes or overlapping regions (in base pairs-bp) are marked below each section. Each transcription unit is shown in different color. Genes encoding T9SS components are depicted in red font. Black vertical arrow shows continuous region (75 bp) between *PG0026* (*porU*) and *PG0027* (*porV*) but the two genes were predicted to transcribe independently. Green arrows indicate operons that were confirmed experimentally (Taguchi et al., 2015; Vincent et al., 2017). Green asterisk denotes proved single transcription unit for the *PG0191-PG0192-PG0193* genes (in *P. gingivalis* ATCC33277 strain), however co-transcription of preceding the *PG0190* gene (17 bp interval) was not investigated (Taguchi et al., 2015). The *PG0809* (*Sov*) gene was re-sequenced and confirmed to consist of the two combined genes *PG0809* and *PG0810*, mis-annotated in W83 genome as separate ORFs (Saiki and Konishi, 2007). A dashed arrow denotes indirect evidence that *PG0809* (*Sov*) and *PG0811* may be co-transcribed. It was shown that sigma factor SigP (regulator of other *por* genes) binds to the region preceding *PG0811* but not the one before *PG0809* (Kadowaki et al., 2016).

Genes encoding T9SS components are scattered around the *P. gingivalis* genome. The exception is a group of five genes, *porP*-*porK*-*porL*-*porM*-*porN*, that are co-transcribed (Vincent et al., 2016). In many other *Bacteroidetes* species, the operon structure of these genes is conserved [databases: STRING (Snel et al., 2000), DOOR (Dam et al., 2007; Mao et al., 2009), ProOpDB (Taboada et al., 2012), OperonDB (Pertea et al., 2009)]. Orthologs of the *porP* gene (*sprP* in some gliding motility bacteria) show the most variation, as the gene can be located in different genomic loci (e.g., *F. johnsoniae Fjoh_3477* vs. *gldK/Fjoh_1853*), and, even if they precede *porK*, they remain as separate transcriptional units (e.g., *C. hutchinsonii sprP/CHU_0170* and *gldK/CHU_0171*; Zhu and McBride, 2014). The rest of the *P. gingivalis* T9SS genes are either single units or predicted to be in 2–5 gene operons (Figure 2) with genes unrelated to T9SS structure and function. In addition, none of the adjacent genes encode T9SS cargo proteins.

### Cytoplasmic and IM components

Presently, there is only one known T9SS-related protein residing entirely in the cytoplasm: PorX (PG0928/PGN_1019). It is a response regulator (RR) of a two-component system (TCS) involved in regulating the expression of several T9SS genes. Its sensor kinase partner, PorY (PG0052/PGN_2001), is an IM-anchored protein containing two transmembrane (TM) helices and a large cytoplasmic domain (~222 aa; Sato et al., 2010; Vincent et al., 2016). Both proteins will be discussed in more detail in the Regulation Section.

Two other essential components of T9SS, PorL (*PG0289 /PGN_1675*) and PorM (*PG0290/PGN_1674*), are also anchored in the IM. PorL possesses two TM helices located between residues 17–48 and 48–74, with both N- and C-termini in the cytoplasm. The precise locations of the helices (the exact amino acids) have not been determined (Vincent et al., 2017). The large cytoplasmic C-terminal domain (~236 residues) interacts *in vitro* with PorX (Vincent et al., 2016); thus it may be involved in regulating T9SS function. Moreover, PorL cytoplasmic domain forms a homotrimer in *E. coli* cells and the full-length protein was found in a complex with PorM both *in vitro* (Gorasia et al., 2016; Vincent et al., 2017) and *in vivo* (Sato et al., 2010). PorM is anchored in the IM by a single TM helix at its N-terminus (between residues 9 and 41), with the remaining residues (475) forming a domain facing the periplasm. In *E. coli* cells, the periplasmic part of PorM dimerizes and interacts with two other core T9SS proteins: PorK and PorN (Vincent et al., 2017). The recombinant periplasmic domain (amino acid residues 36–516) was crystallized, presenting with tetragonal crystals, but automatic model building failed to provide a realistic structure, thus leaving the nature of interactions unknown (Stathopulos et al., 2015). Nevertheless, a possible function for PorL/PorM, apart from the regulatory implications for PorL, has been suggested.

It was proposed that the two proteins form an energy transducer complex to provide energy for T9SS assembly and substrate translocation. The idea came from *F. johnsoniae*, which utilizes a proton-motive force for gliding motility (Nakane et al., 2013; Gorasia et al., 2016). It was further noted that the hydrophobic TM helixes of GldL (PorL ortholog), PorL, and PorM possess conserved glutamate residues characteristic of known energy transducers (Shrivastava et al., 2013; Vincent et al., 2017). These assumptions need experimental verification; nevertheless, they are compatible with mechanisms used by other secretion systems to provide the energy needed to drive substrate transport such as hydrolysis of ATP, proton-motive force, low-energy assembly, and entropy gradient (Costa et al., 2015).

### Periplasmic components

Four T9SS proteins are located in the periplasm: PorN (PG0291/PGN_1673), PorK (PG0288/PGN_1676), PorW (PG1947/PGN_1877), and PG1058/PGN_1296. All but one (PorN) are predicted or proven to be lipoproteins associated with membranes (Sato et al., 2010). PorW is the least investigated protein among the periplasmic elements of *P. gingivalis* secretion. Experimental work on PorW has only been performed on the *F. johnsoniae* PorW ortholog, SprE (Fjoh_1051), which is a predicted lipoprotein that localizes to a membrane fraction (most likely the OM). A mutant with a deleted *sprE* gene exhibits phenotypes in gliding bacteria typical of other T9SS function-deficient mutants, such as non-spreading colonies, defective gliding, and blocked secretion of chitinase (Rhodes et al., 2011; Kharade and McBride, 2015). Its subcellular localization and the effects of its mutation on the secretory/gliding phenotype suggest that SprE/PorW is yet another structural component of T9SS.

PG1058 is a multidomain protein necessary for T9SS function. The phenotype of *P. gingivalis* with an inactivated *PG1058* gene is typical of other T9SS mutants: colonies on blood agar lack pigmentation and inactive, unprocessed gingipains accumulate in the periplasm. The PG1058 protein is anchored by its lipid modification to the periplasmic surface of the OM. The predicted structure suggests the presence of four structural domains: a tetratricopeptide repeat (TPR) domain, a β-propeller domain, a carboxypeptidase regulatory domain-like fold (CRD), and an OmpA_C-like putative peptidoglycan-binding domain. TPR and β-propeller domains are involved in protein-protein interactions; hence, together with the *PG1058* mutant phenotype, it is plausible that PG1058 supports the T9SS translocon structure (Heath et al., 2016). Further, experiments are needed to verify this hypothesis.

PorN is a periplasmic protein that forms dimers *in vitro* and has the propensity to interact both *in vitro* and *in vivo* with IM protein PorM and periplasmic lipoprotein PorK (Gorasia et al., 2016; Vincent et al., 2017). The nature of the interaction with PorK is interesting, as both proteins form a ring-shaped structure with an external and internal diameter of 50 and 35 nm, respectively. It was proposed that they form a large complex in which PorN interacts in an almost 1:1 fashion (32–36 total subunits) with the PorK lipoprotein. The ring structure is anchored into the OM through the fatty acids of PorK. Consistent with detected interactions, PorN has a crucial role in stabilizing both PorL–PorM and PorN–PorK complexes, as deletion of the *porN* gene resulted in the degradation of PorL, PorM, and PorK in *P. gingivalis* cells. By contrast, deletion of either *porL* or *porM* does not interfere with the stability of the PorN/K complex (Gorasia et al., 2016).

Further, studies on PorK,L,M,N interactions suggest the existence of a PorK_2_L_3_M_2_N_2_ complex that likely oligomerizes to form a superstructure with a final molecular mass of over 1.2 MDa (Gorasia et al., 2016; Vincent et al., 2017). Such a large complex was originally reported by Sato and colleagues, who identified all four proteins in a single spot on a blue-native electrophoresis gel (Sato et al., 2010). However, additional elements of the complex were recently identified: PG0189 and PorP (PG0287/PGN_1677). Because they are predicted to be integral OM β-barrel proteins, they are discussed in more detail in the following section.

### OM and surface components

The vast majority of T9SS components are confined to the OM. In addition to the peripheral OM-associated and periplasmic proteins delineated above, seven others (Sov, PorQ, PorP, PorT, PorV, PG0189, and PG0534) are predicted to be integral OM β-barrel proteins. Furthermore, two proteins, PorU and PorZ, are associated with the bacterial surface. In addition, PG0192 was found in a membrane fraction, but its association with the OM needs further verification.

PorT and Sov were the first proteins found to be essential for *P. gingivalis* protein secretion, and the discovery led to intense research on T9SS (see Discovery Section; Sato et al., 2005, 2010; Saiki and Konishi, 2007). Despite this, we still know very little about the structure and function of these proteins a decade later. PorT is predicted to have eight anti-parallel, membrane-traversing β-strands, with four large loops facing the environment, and this topology has been experimentally confirmed (Nguyen et al., 2009). Sov was also described as an integral OM protein with its C-terminal region likely exposed to the extracellular milieu (Saiki and Konishi, 2007, 2010b). However, the precise roles of both proteins in T9SS structure and function remain unknown. Even less information is available concerning PorQ (PG0602/PGN_0645) as a T9SS component (Sato et al., 2010). In the genome annotation, it is described as a hypothetical protein with a β-barrel structure belonging to the porin superfamily (Nelson et al., 2003); thus it is assumed to localize to the OM.

Similarly, little is known about PG0534/PGN_1437 as a protein essential for T9SS function (Saiki and Konishi, 2010a). Interestingly, *PG0534* is upregulated in human gingival epithelial cells, suggesting its contribution to *P. gingivalis* eukaryotic cell invasion and/or intracellular survival (Park et al., 2004). *In silico* predictions run on the RaptorX server (Kallberg et al., 2012) modeled PG0534 as a β-barrel OM protein, with the pyochelin OM receptor FptA from *Pseudomonas aeruginosa* (Cobessi et al., 2005) as the best template (PDB: 1xkwA; *p*-value: 1.82e-23).

The next T9SS OM component, PG0192/PGN_300 (annotated as an OmpH-like protein), was found in the total membrane fraction. Due to its 17 kDa molecular mass, the protein is referred to as Omp17 (Taguchi et al., 2015). The best template prediction by the RaptorX server is a putative OM chaperone (OmpH-like) from *Caulobacter crescentus* (PDB: 4kqtA; *p*-value: 6.78e-04). The phenotypic effects of *omp17* mutation are typical of other T9SS-defective mutants but with an interesting exception. The mutant is still able to secrete unprocessed T9SS cargo proteins, including pro-gingipains and CPG70, which accumulate in the periplasm in other secretion mutants (Taguchi et al., 2015). Of note, in the wild-type *P. gingivalis*, T9SS cargos remain attached to the bacterial surface through anionic lipopolysaccharide (A-LPS) anchoring (Shoji et al., 2002; Shoji and Nakayama, 2016). This modification is added by the surface-located PorU protein (Gorasia et al., 2015; for more details see the Mechanism Section). Taguchi and colleagues showed that A-LPS synthesis in the *omp17* mutant was not affected, suggesting the impairment of PorU function. Consistent with that, PorU was not detected in the *omp17*^−^ cell envelope fraction, but was found in the cytoplasm/periplasm fraction. Moreover, the *omp17* mutant was less virulent than the wild type in the mouse subcutaneous model, which is consistent with the lack of gingipain activity (Taguchi et al., 2015).

As previously mentioned, PG0189 and PorP (a part of the *porPKLMN* operon) were detected in association with the PorKLMN complex. Specifically, a periplasmic loop of PG0189 interacts with both PorK and PorN, as shown by cross-linking experiments. Due to its low abundance, PG0189 is proposed to play an accessory role in secretion (Gorasia et al., 2016). The nature of the interaction of PorP with PorK and PorM is still enigmatic. The proteins co-precipitate *in vitro*; however, all tested proteins were produced in *E. coli* cells, and, so far, have not been detected in the native complex (Vincent et al., 2017).

Currently, the only OM β-barrel protein with an assigned function is PorV (PG0027/PGN_0023/LptO). The PorV-mutated strain retains inactive, unprocessed gingipains in the periplasm (Ishiguro et al., 2009) and fails to O-deacylate LPS, which might be a necessary step in post-translational processing during the secretion of cargo proteins (Chen et al., 2011; Glew et al., 2012). Yet another study indicated that PorV interacts *in vivo* with PorU (PG0026/PGN_0022), and it was proposed that PorV serves as an OM anchor for PorU (Saiki and Konishi, 2014). Indeed, PorU localizes to the surface of *P. gingivalis* cells and is involved in T9SS cargo processing (see the next section; Glew et al., 2012; Gorasia et al., 2015). Despite this relative abundance of knowledge on PorV, it remains unknown whether PorV is directly involved in LPS processing or if it is only an accessory protein for an unknown LPS O-deacylase. The secretion-deficient phenotype of the PorV mutant might be related to the lack of PorU immobilization on its surface.

The last known component of T9SS is a surface-located PorZ protein (PG1604/PGN_0509) recently characterized by our group (Lasica et al., 2016). The non-pigmented phenotype of the PorZ-mutant strain and its accumulation of unprocessed, inactive gingipains confirmed that PorZ is essential for the system. Interestingly, it was shown (through proteomics and mutagenesis studies) that PorZ is itself a cargo of T9SS and has the conserved C-terminal domain (CTD) (Glew et al., 2014; Lasica et al., 2016). The CTD works as a signal, directing T9SS cargo proteins to the OM translocon (see the next section; Shoji et al., 2011). However, unlike other cargos, the CTD of PorZ is not cleaved off upon secretion and the protein is not anchored in the OM in the same manner as other secreted proteins (Lasica et al., 2016). This phenomenon was observed for only one other protein, PorU, which is also both a functionally essential element and a cargo of T9SS (Glew et al., 2012). PorZ is currently the sole Por protein with a solved atomic structure. It is composed of two large β-propeller domains and a CTD, conforming to canonical β-sandwich architecture (de Diego et al., 2016; Lasica et al., 2016). Although the precise role of PorZ remains to be revealed, β-propeller domains are a good platform for protein-protein interactions and provide binding areas for small molecules (e.g., saccharides; Hunt et al., 1987; Zhang et al., 2014). Considering the structure and processing, we hypothesize that, like PorU, PorZ may be involved in post-translational maturation of T9SS cargo proteins during their translocation across the OM.

## Mechanism of secretion

Protein secretion using T9SS is a two-step process. First, the cargo proteins are guided by a classical signal peptide to the Sec machinery in the IM. During translocation, the signal peptide is cleaved off by type I signal peptidase, and the cargo is released into the periplasm. Although, the Sec pathway has not been experimentally analyzed in *P. gingivalis*, the screening of *Bacteroidetes* genomes confirmed that the system is mostly conserved (McBride and Zhu, 2013). In the periplasm, transported proteins fold into a stable conformation, as indicated from the accumulation of their soluble forms in the periplasm of T9SS secretory mutants. Whether the cargo proteins require a chaperone(s) to assist in folding and/or guiding them to the OM translocon is still unknown.

A common feature of all T9SS cargo proteins is the conserved CTD that targets T9SS cargo proteins to the OM translocon. The function of the CTD was first recognized while studying the secretion and processing of the RgpB (PG0506/PGN_1466) gingipain. The protein without the C-terminal Ig-like domain of 72 amino acid residues was not secreted, but accumulated in the periplasm of the mutated *P. gingivalis* strain in its truncated form (Seers et al., 2006). A parallel study confirmed this observation, showing that the integrity of the CTD is essential for RgpB secretion, as even truncating the C-terminal by two residues hinders transport across the OM. The same effect is caused by mutating the highly conserved residues at the C-terminus of the CTD (Nguyen et al., 2007). The elegant follow-up investigations with CTDs from different *P. gingivalis* T9SS cargo proteins (HBP35/PG0616/PGN_0659, CPG70/PG0232/PGN_0335, P27/PG1795/no PGN, and RgpB) genetically fused to GFP found that GFP was secreted and post-translationally modified by *P. gingivalis* in the same way as the native T9SS cargos. The secretion/modification signal was narrowed down to the last 22 residues of the CTD domain (Shoji et al., 2011), and proteomic analysis revealed cleaved CTDs in the culture medium (Veith et al., 2013).

Taken together, these findings suggested the existence of a C-terminal-sorting peptidase responsible for the proteolytic removal of the CTD during the cargos' translocation across the OM. The postulated sortase was identified in *P. gingivalis* as PorU, a surface-located cysteine peptidase that shares significant sequence similarity with gingipains (see previous section; Glew et al., 2012). Analysis of the cleavage sites of T9SS cargos in *P. gingivalis* revealed a PorU preference toward polar or acidic amino acid residues (Ser, Thr, Asn, Asp) at the carbonyl site (P1′ position) and small amino acid residues (such as Gly, Ser, Ala) at the amide site (P1 position; Glew et al., 2012; Veith et al., 2013). This low specificity of PorU was confirmed when the amino acids surrounding the cleavage site (P1–P1′) in RgpB were mutated. Of note, this did not affect the secretion of the gingipain (Zhou et al., 2013).

### Secretion signal for T9SS substrates is embedded in the secondary structure

Bioinformatic analysis of 21 fully sequenced genomes from the *Bacteroidetes* phylum revealed the presence of 663 predicted CTD-containing proteins (Veith et al., 2013). Alignment of the amino acid sequence of identified CTDs revealed up to five conserved sequential motifs (A–E) in different T9SS cargo proteins (Seers et al., 2006; Nguyen et al., 2007; Slakeski et al., 2011). Out of these, two sequential motifs, PxGxYVV and KxxxK, that reside in the last 22 amino acids of CTDs are the most conserved. This conservation is consistent with this fragment being sufficient for secretion in *P. gingivalis* (Shoji et al., 2011; Veith et al., 2013). Cumulatively, however, the limited sequence identity of CTDs suggests that the signal recognized by the T9SS machinery is not imprinted in the amino acid sequence but is formed by a specific fold of the CTD. This contention was confirmed by the atomic structure of the CTD from two *P. gingivalis* T9SS cargo proteins: RgpB and PorZ (de Diego et al., 2016; Lasica et al., 2016). Their CTDs consist of seven β-strands of similar length, generating a compact, sandwich-like fold typical of an immunoglobulin-superfamily (IgSF) domain. Analysis of the CTD of RgpB revealed a propensity of the protein to dimerize by swapping the last β-strand (de Diego et al., 2016). Of note, the last two β-strands overlap perfectly with the 22 amino acid residues essential for secretion of CTD proteins (Shoji et al., 2011). Despite the differences within the loops and the low amino acid sequence similarity, the PorZ-derived CTD structure is topologically equivalent to that of RgpB. This conclusion likely extends to the majority of identified CTDs, which share the fold of the IgSF domain. Therefore, the tertiary structure of the CTD, especially its two terminal β-strands, likely contains the signal recognized by the T9SS translocon (Lasica et al., 2016).

### Secretion-associated modifications of T9SS cargo proteins

The characteristic feature of T9SS function is the retention of cargo proteins on the bacterial surface. SDS-PAGE analysis of OM-associated proteins produced diffuse bands about 20 kDa larger than that predicted from the primary structure of T9SS-secreted proteins (Veith et al., 2002). The difference is due to the presence of an A-LPS (Paramonov et al., 2005; Rangarajan et al., 2008) covalently attached to the cargo proteins imbedded into the OM, as indicated by western blot using specific antibodies (Abs). By contrast, the molecular mass of proteins accumulating in the periplasm of secretion mutants correlates well with the predicted molecular mass, and the proteins have no reactivity with anti-A-LPS Abs (Shoji et al., 2014). In addition, electron microscopy revealed that CTD-containing proteins (especially gingipains) form the electron-dense surface layer (EDSL) encapsulating *P. gingivalis* cells (Chen et al., 2011). Gorasia et al. (2015) found that the *wbaP* (PG1964/PGN_1896) mutant of *P. gingivalis*, which is defective in A-LPS synthesis, completely lacks the EDSL and releases T9SS cargos in soluble form into culture fluid. The proteins lack CTDs, suggesting normal PorU sortase activity, but are not A-LPS modified and therefore cannot be incorporated into the OM (Gorasia et al., 2015).

The mechanism of A-LPS attachment to CTD-containing proteins during secretion by T9SS is still unknown. The analysis of CTD proteins isolated from the growth media of the *wbaP* mutant revealed that peptides/amino acids derived from growth medium or glycine (if added in excess to the broth) were added to the proteins' C-termini via peptide bond. On the other hand, a 648 Da linker attached to C-termini by an isopeptide bond was identified in CTDs derived from the wild-type *P. gingivalis* strain (Gorasia et al., 2015). Such modification is reminiscent of a sortase-like mechanism of protein binding to peptidoglycan in Gram-positive bacteria. Sortases are cysteine proteases (C60 family) that have a catalytic Cys/His dyad, characteristic for many cysteine proteases, and possess a conserved Arg residue essential for sorting activity (Marraffini et al., 2004). This Arg is absent in gingipains, but is found in PorU sortase (Gorasia et al., 2015). All these findings suggest that PorU is a sortase, the first identified among Gram-negative bacteria. It cleaves the CTD and simultaneously attaches the A-LPS moiety to the newly generated C-terminus of a cargo protein via a linker of unknown structure. In this context, the T9SS mechanism resembles the covalent attachment of proteins to the cell wall in Gram-positive bacteria such as *S. aureus* (Schneewind and Missiakas, 2012).

## Regulation

Essential T9SS genes, including *porT, porV, sov, porP, porK, porL, porM*, and *porN*, are regulated at the transcriptional level by a signaling pathway composed of the PorXY two-component system (TCS) and an extracytoplasmic function (ECF) sigma factor (SigP/PG0162/PGN_0274; Kadowaki et al., 2016). In contrast to the majority of TCSs, in which the components are encoded within the same operon, the *porX* and *porY* genes occur at separate loci within the *P. gingivalis* chromosome. Despite this unusual genomic organization, the activation of the PorXY TCS is canonical. PorY has a modular architecture typical for a histidine kinase (HK) and undergoes autophosphorylation at His193, as shown by radiolabeled [^32^P-γ]ATP. The phosphate group is then transferred to the conserved Asp58 residue in the receiver domain of PorX, which functions as the response regulator (RR). To compensate for the lack of a DNA-binding domain in the RR, PorX interacts with SigP, which directly binds the promotor regions of T9SS genes. The SigP protein level is very low in the *porX*-deletion mutant, suggesting a stabilizing function for PorX on SigP (Kadowaki et al., 2016). Disruption of the PorXY TCS results in the dysfunction of T9SS, which manifests as the decrease of Rgp and Kgp activity, as well as the impaired processing of gingipains (Sato et al., 2010).

PorX can also modulate the T9SS architecture directly by interacting with the cytoplasmic domain of PorL (Vincent et al., 2016). The N-terminal domain of PorX is similar to RRs belonging to the CheY family, which are involved in chemotaxis. After phosphorylation, the CheY protein binds to the C-ring of flagella, which changes the direction of flagellar movement (Roman et al., 1992; Sagi et al., 2003). Due to the fact that T9SS was proposed to be a rotary apparatus enabling the rotary movement of SprB adhesin in gliding bacteria (Shrivastava et al., 2015), it has been speculated that the PorX mechanism might be similar to that of CheY (Vincent et al., 2016). However, its role in *P. gingivalis* cells will likely be different as this bacterium is non-motile.

There are other studies reporting the changes in a T9SS protein's expression profile under specific circumstances. In a PorZ-deletion strain, some of the T9SS genes (including *porT, porV*, and *porN*), together with genes encoding CTD-cargo peptidases (RgpB, Kgp, and CPG70), are upregulated, whereas the expression of other T9SS genes (such as *porQ, porW, sov*, and *porU*) is not changed (Lasica et al., 2016). Additionally, the gliding motility protein GldN (orthologous of *P. gingivalis* PorN) of *Flavobacterium psychrophilum* is significantly upregulated under iron-limited growth conditions and *in vivo* (LaFrentz et al., 2009). The expression of T9SS proteins must be strictly regulated to fine-tune the energy-absorbing secretion of proteins into the environment. However, a precise environmental signal has not been identified and our knowledge about T9SS regulation is still limited.

## Protein effectors in *P. gingivalis*

Only a few secretion systems are dedicated to carrying a single cargo protein; examples are HlyA in *E. coli* and HasA in *S. marcescens* for T1SS (Kanonenberg et al., 2013), and PulA in *K. oxytoca* and LT toxin in *E. coli* for T2SS (Rondelet and Condemine, 2013). The majority of secretion systems translocate many proteins of similar or diverse functions [e.g., T3SS; Gaytan et al., 2016]. In many respects, T9SS is one of the most robust secretion systems, which, in *P. gingivalis* alone, facilitates secretion of up to 35 cargos bearing the CTD (see Table 2), many of which are implicated in bacterial pathogenicity. In fact, experiments conducted to characterize the important virulence factors (the gingipains RgpA, RgpB, and Kgp) contributed to the discovery of T9SS. Below, we briefly describe only the most important cargos from the point of view of *P. gingivalis* virulence. References to other cargo proteins can be found in Table 2.

**Table 2:** **T9SS cargo proteins**.

***Porphyromonas gingivalis**^a^*					
**Locus Tag**		
**W83 NC_002950.2**		**ATCC33277 NC_010729.1**	**Protein accession number**	**Protein description**	**References**
PG_RS00120	PG0026	PGN_0022	WP_005874469.1	PorU; surface C-terminal sortase	Glew et al., 2012; Veith et al., 2013; Gorasia et al., 2015
PG_RS00835	PG0182	PGN_0291	WP_010955943.1	Mfa5; VWA domain-containing protein [von Willebrand factor (vWF) type A domain]	Hasegawa et al., 2016
PG_RS00840	PG0183	no PGN	WP_043876389.1	Hypothetical protein containing VWA domain identical to that in PG0182 (circa 430 residues); lipoprotein	Found only by proteomic analysis^a^
PG_RS01060	PG0232	PGN_0335	WP_005873522.1	CPG70; zinc carboxypeptidase	Veith et al., 2004; Shoji et al., 2011; Zhou et al., 2013
PG_RS01560	PG0350	PGN_1611	WP_005873799.1	Internalin; hypothetical protein; leucine-rich repeats (x8)	Found only by proteomic analysis^a^
PG_RS01820	PG0410	no PGN	WP_005873803.1	Hypothetical gingipain-like peptidase C25	
PG_RS01825	PG0411	PGN_1556	WP_010956006.1	T9SS C-terminal target domain-containing protein	Found only by proteomic analysis^a^
PG_RS02195	PG0495	PGN_1476	WP_010956042.1	T9SS C-terminal target domain-containing protein	Found only by proteomic analysis^a^
PG_RS02240	PG0506	PGN_1466	WP_010956050.1	RgpB; arginine specific gingipain B, cysteine protease	Pike et al., 1994; Seers et al., 2006; Guo et al., 2010; de Diego et al., 2016
PG_RS02455	PG0553	PGN_1416	WP_010956068.1	PepK; lysine specific serine endopeptidase	Sato et al., 2013; Nonaka et al., 2014; Veith et al., 2014
PG_RS02700	PG0611	PGN_0654	WP_043876409.1	Hypothetical protein	Found only by proteomic analysis^a^
PG_RS02710	PG0614	PGN_0657	WP_005874506.1	Hypothetical protein	Found only by proteomic analysis^a^
PG_RS02720	PG0616	PGN_0659	WP_005874521.1	HBP35 (hemin binding protein 35)	Shoji et al., 2010, 2011
PG_RS02765	PG0626	no PGN	WP_005874512.1	T9SS C-terminal target domain-containing protein	Found only by proteomic analysis^a^
PG_RS02890	PG0654	PGN_0693	WP_005873571.1	T9SS C-terminal target domain-containing protein	Found only by proteomic analysis^a^; Glew et al., 2012
PG_RS03370	PG0769	PGN_0795	WP_010956121.1	Fibronectin; hypothetical protein^b^	Found only by proteomic analysis^a^; Sato et al., 2013
PG_RS03450	PG0787	PGN_0810	WP_005873930.1	T9SS C-terminal target domain-containing protein^c^	Found only by proteomic analysis^a^
PG_RS04535	PG1030	PGN_1321	WP_005874101.1	T9SS C-terminal target domain-containing protein	Found only by proteomic analysis^a^
PG_RS05835	PG1326	PGN_1115	WP_005875446.1	Hemagglutinin	Found only by proteomic analysis^a^
PG_RS06055	PG1374	PGN_0852	WP_005874331.1	T9SS C-terminal target domain-containing protein, leucine-rich repeats (x7)	Found only by proteomic analysis^a^; Glew et al., 2012
PG_RS06255	PG1424	PGN_0898	WP_005873463.1	PPAD; peptidylarginine deiminase	Sato et al., 2013; Koziel et al., 2014; Goulas et al., 2015
PG_RS06260	PG1427	PGN_0900	WP_005873781.1	Periodontain; peptidase C10; PrtT-related	Nelson et al., 1999
PG_RS06835	PG1548	PGN_0561	WP_043876505.1	PrtT; cystein protease (domain peptidase C10)	Madden et al., 1995; Gorasia et al., 2015
PG_RS07070	PG1604	PGN_0509	WP_010956350.1	PorZ; surface B-propeller protein	Lasica et al., 2016
PG_RS07920	PG1795	PGN_1770	WP_005874140.1	Hypothetical protein	Found only by proteomic analysis^a^
PG_RS07930	PG1798	PGN_1767	WP_005874135.1	T9SS C-terminal target domain-containing protein	Found only by proteomic analysis^a^
PG_RS08090	PG1837	PGN_1733	WP_043876452.1	HagA (hemagglutinin A, 8 HA domains)	Shi et al., 1999; Glew et al., 2012; Saiki and Konishi, 2014
PG_RS08105	PG1844	PGN_1728	WP_043876454.1	Kgp; lysine specific gingipain, cysteine protease	Pike et al., 1994; Veith et al., 2002
PG_RS08700	PG1969^d^	no PGN	WP_010956456.1	T9SS C-terminal target domain-containing protein	Found only by proteomic analysis^a^
PG_RS08940	PG2024	PGN_1970	WP_010956476.1	RgpA; arginine specific gingipain A; cysteine protease	Pike et al., 1994; Veith et al., 2002; Glew et al., 2012
PG_RS09310	PG2100	no PGN	WP_005873768.1	T9SS C-terminal target domain-containing protein; TapC	Kondo et al., 2010; Sato et al., 2013
PG_RS09320	PG2102	PGN_0152	WP_005873754.1	T9SS C-terminal target domain-containing protein; TapA	Kondo et al., 2010; Glew et al., 2012; Sato et al., 2013
PG_RS09640	PG2172	PGN_0123	WP_005874973.1	Hypothetical protein	Found only by proteomic analysis^a^; Glew et al., 2012
PG_RS09755	PG2198	PGN_2065	WP_005874281.1	Hypothetical protein; peptidase	Found only by proteomic analysis^a^
PG_RS09850	PG2216	PGN_2080	WP_010956525.1	Hypothetical protein	Found only by proteomic analysis^a^; Glew et al., 2012
***Tannerella forsythia* ATCC43037**					
Tanf_03370			WP_046824918.1	TfsA (surface layer protein A), classical CTD	Tomek et al., 2014
Tanf_03375			WP_046824919.1	TfsB (surface layer protein B), classical CTD	Tomek et al., 2014
Tanf_04820			WP_046825062.1	BspA, cell surface antigen, leucine rich protein, classical CTD	Veith et al., 2009; Friedrich et al., 2015
Tanf_06225			WP_046825275.1	Forsilysin, metalloprotease, KLIKK-type CTD	Narita et al., 2014
Tanf_00450			WP_070098098.1	Mirolysin, metalloprotease, KLIKK-type CTD	Karim et al., 2010; Ksiazek et al., 2015a,b; Koneru et al., 2017
Tanf_06550			D0EM77.2	Karilysin, metalloprotease, KLIKK-type CTD	Karim et al., 2010; Narita et al., 2014; Ksiazek et al., 2015a; Koneru et al., 2017
Tanf_00440			AIZ49398.1	Mirolase, serine protease, KLIKK-type CTD	Karim et al., 2010; Ksiazek et al., 2015a,b; Koneru et al., 2017
Tanf_09450, Tanf_06530 (not merged in one contig)			AKG97061.1	Miropsin-1, serine protease, KLIKK-type CTD	Ksiazek et al., 2015b
Tanf_06530			WP_046825306.1	Miropsin-2, serine protease KLIKK-type CTD	Narita et al., 2014
***F. johnsoniae*** **UW101**^e^					
Fjoh_4555			WP_012026520.1	ChiA, chitinase	Rhodes et al., 2010; Kharade and McBride, 2014
Fjoh_0979			WP_012023065.1	SprB, surface adhesin, necessary for gliding motility	Rhodes et al., 2010; Shrivastava et al., 2013
Fjoh_0808			WP_052295174.1	RemA, mobile surface adhesin, necessary for gliding motility	Shrivastava et al., 2012, 2013

a *All P. gingivalis cargo proteins excluding PG0410 (no PGN) and PG1548 (PGN_0561) were originally found by Veith et al. (2013)*.

b *PG0769 (PGN_0795) processing is unclear. Protein is devoid of N-terminal cleavage signal for periplasm transport (searched with SignalP and LipoP servers) as well as T9SS CTD domain*.

c *PG0787 (PGN_0810) is a very small peptide (80 aa) devoid of N-terminal cleavage signal for periplasm transport (searched in SignalP and LipoP servers), however its last 66 aa constitute a classical T9SS CTD domain*.

d *PG1969 processing is unclear. Protein is devoid of N-terminal cleavage signal for periplasm transport (searched with SignalP and LipoP servers) but contains T9SS CTD domain*.

e *Proteins listed are the best studied among other identified T9SS cargos of F. johnsoniae. For more information please see Kharade and McBride (2015)*.

### Gingipains and CPG70

There are three enzymes collectively termed gingipains: RgpA (PG2024/PGN_1970), RgpB (PG0506/PGN_1466), and Kgp (PG1844/PGN_1728). They are cysteine proteases that hydrolyze peptide bonds at the carboxyl group of arginine (RgpA/B: Arg–Xaa) or lysine residues (Kgp: Lys–Xaa; Pike et al., 1994). They are exported into the periplasm as inactive zymogens, with the N-terminal prodomain (NTP) functioning as a chaperone and maintaining the latency of the proteases (Mikolajczyk et al., 2003; Pomowski et al., 2017). After folding in the periplasm, they are transported to the bacterial surface, where they are subjected to extensive post-translational processing. The CTD is cleaved by PorU sortase during translocation, with the concomitant covalent attachment of A-LPS via an isopeptide bond to the newly formed carbonyl group (Glew et al., 2012; Gorasia et al., 2015). Then, the OM-anchored gingipains activate themselves by cleaving off the NTP. For RgpB, this is the end of processing, but the polypeptide chains of RgpA and Kgp are further fragmented to form a large, non-covalent complex of catalytic and hemagglutinin domains on the bacterial surface (Bhogal et al., 1997; Veith et al., 2002; Sztukowska et al., 2012). The activation and further processing are still not well-understood, and, in addition to trans- and cis-autoproteolysis, they also involve the removal of the C-terminal Arg and Lys residues by the Arg/Lys-specific carboxypeptidase CPG70 (PG0232/PGN_0335; Chen et al., 2002). Interestingly, CPG70 is a T9SS substrate itself (Veith et al., 2004; Zhou et al., 2013). Of note, the retention of gingipains, CPG70, and other T9SS cargos on the bacterial surface depends on the synthesis of A-LPS. The *P. gingivalis* strain HG66, which lacks the activity of an enzyme in the A-LPS synthesis pathway, secretes soluble gingipains into the media (Pike et al., 1994; Shoji et al., 2014; Siddiqui et al., 2014).

Gingipains are the most powerful weapon within the *P. gingivalis* arsenal of virulence factors, as they are responsible for nearly 85% of the total proteolytic activity (Potempa et al., 1997). They are responsible for a variety of pathogenic functions such as colonization, nutrition, neutralization of host defenses, and alteration of the inflammatory response, which all lead to massive oral tissue destruction called periodontitis during prolonged infection (reviewed in Guo et al., 2010; Bostanci and Belibasakis, 2012; Hajishengallis, 2015). However, gingipains are not only directed against host proteins, but are also involved in processing other *P. gingivalis* proteins [e.g., long fimbriae (FimA)] (Nakayama et al., 1996; Xu et al., 2016). Interestingly, gingipains' activities rely on their local concentration, resulting in either activation of some pathways at low concentrations (specifically human complement) or destroying them upon accumulation (Krauss et al., 2010). Moreover, despite the cleavage specificity to a single C-terminal Arg or Lys residue, they can act in a precise and fastidious manner or as unlimited shredders (Potempa et al., 2000; Sroka et al., 2001; Goulet et al., 2004).

Considering the broad range of activities combined with cell surface localization, it is not surprising that gingipains are a tempting target for designing periodontitis treatments as well as preventive strategies (inhibitors and vaccines; Olsen and Potempa, 2014; Inaba et al., 2016; Wilensky et al., 2016).

### Porphyromonas peptidylarginine deiminase (PPAD)

Porphyromonas peptidylarginine deiminase (PPAD), encoded by PG1424/PGN_0898, is a unique enzyme among prokaryotes. It is the first and only bacterial peptidylarginine deiminase (PAD) identified, and, moreover, its presence is limited to a single species: *P. gingivalis* (McGraw et al., 1999; Gabarrini et al., 2015).

PADs are well-described eukaryotic enzymes functioning in vertebrates as post-translational modifiers of proteins. Specifically, they citrullinate internal arginine residues, which changes the fold, function, and half-life of proteins and peptides (Vossenaar et al., 2003; Gyorgy et al., 2006). Dysregulation of this process, particularly the accumulation of citrullinated proteins, leads to inflammatory disorders and has been associated with numerous diseases such as Alzheimer's disease, multiple sclerosis, psoriasis, fibrosis, cancer, and rheumatoid arthritis (RA) (Vossenaar et al., 2003; Chang and Han, 2006; Baka et al., 2012; Gudmann et al., 2015). The latter develops through an autoimmune response against citrullinated proteins and is enhanced by a combination of environmental and genetic factors (MacGregor et al., 2000; McInnes and Schett, 2011). Currently, periodontal disease is an acknowledged RA risk factor, and the discovery of PPAD uncovered a missing mechanistic link between the two illnesses (Wegner et al., 2010; Koziel et al., 2014; Quirke et al., 2015; Laugisch et al., 2016).

PPAD was identified as a T9SS substrate through proteomics studies of a *porT* mutant (Sato et al., 2013); however, the enzyme was characterized mostly in relation to its function rather than secretion. It citrullinates C-terminal arginine residues in a calcium-independent manner, whereas eukaryotic PADs are Ca^2+^-dependent (Takahara et al., 1986; McGraw et al., 1999; Abdallah et al., 2007; Wegner et al., 2010; Bielecka et al., 2014). Moreover, the C-terminal specificity of PPAD plays into the cleavage activities of RgpA/B (after Arg), which greatly enlarge the pool of citrullinated substrates from both bacterial and host origins as gingipains cleave numerous human proteins (Guo et al., 2010). Gingipain-null mutants (RgpA/B) are almost devoid of endogenous citrullination (Wegner et al., 2010). Furthermore, even the presence of PPAD (not only its activity) may elevate anti-citrullination immune responses, as it undergoes autocitrullination. Only this form triggers specific Abs in mice and was recognized by RA patients' sera (reviewed in Koziel et al., 2014).

Analysis of PPAD structure revealed that the enzyme is composed of four elements: a profragment, a catalytic domain (CD), an IgSF domain, and a CTD, resembling domains observed in gingipains. The CD has a flat 5-fold α/β-propeller architecture and includes a catalytic triad (C^351^-H^236^-N^297^) also conserved in human PADs (Goulas et al., 2015; Montgomery et al., 2016). The crystal structure of substrate-free and substrate-bound forms confirmed that PPAD is efficient in accommodating and processing C-terminally situated Arg residues regardless of total chain length (peptide or protein; Goulas et al., 2015).

The surface location of PPAD and the availability of its detailed structure, combined with its important role in two prevalent human diseases (periodontitis and RA), should make PPAD a good target for therapeutic strategies; however, no such experiments have been reported.

## T9SS in *T. forsythia*

The mechanism of protein secretion by T9SS was mostly studied in *P. gingivalis* and gliding bacteria. Apart from a different subset of secreted proteins reflecting bacterial habitats, the mechanism of action is the same. Briefly, T9SS cargo proteins are directed to the T9SS machinery by the CTD, which is removed during secretion. Then, secreted proteins may be modified and attached to the surface by A-LPS (*P. gingivalis*), stay associated with the cell through polysaccharides, or be released (gliding bacteria; McBride and Nakane, 2015; Nakayama, 2015). However, analysis of T9SS in another member of the red complex, *T. forsythia*, revealed some interesting differences.

*T. forsythia* is covered with a two-dimensional crystalline surface (S-) layer that is thought to function as a protective coat, working as an external sieve and ion trap (Sleytr and Beveridge, 1999; Messner et al., 2010). It also mediates adhesion and subsequent invasion into human gingival epithelial cells (Sakakibara et al., 2007) and delays recognition of the bacterium by the host innate immune system (Sekot et al., 2012). The S-layer is composed of the glycosylated proteins TfsA (Tanf_03370) and TfsB (Tanf_03375). Deleting *porU* (Tanf_02580), *porT* (Tanf_10520), *sov* (Tanf_04410), or *porK* (Tanf_02360) results in the lack of an S-layer, which can be observed by transmission electron microscope (Narita et al., 2014; Tomek et al., 2014). In those mutants, both components of the S-layer are trapped within the periplasm, but, unlike in *P. gingivalis*, they are modified by O-glycosylation through the addition of multiple copies of a complex oligosaccharide using a general glycosylation pathway operating in *Bacteroidetes* (Coyne et al., 2013; Posch et al., 2013; Tomek et al., 2014). Nevertheless, TfsA and TfsB trapped in the periplasm are much smaller than both proteins in the wild-type cells, indicating that, upon secretion, both proteins are modified by a second glycan attachment in a manner different than O-glycosylation. It is speculated that, as in *P. gingivali*s, it could be a variant of LPS (Tomek et al., 2014).

T9SS cargo proteins in *T. forsythia* have two different types of CTD. The “classical” CTD associated with proteins from other *Bacteroidetes* species is found in TfsA, TfsB, and leucine rich protein BspA (Veith et al., 2009; Tomek et al., 2014). By contrast, a family of six proteases, three metalloproteases (karilysin, mirolysin, and forsilysin) and three serine proteases (mirolase, miropsin-1, and miropsin-2), bear a nearly identical CTD that shares very limited sequence similarity with the classical CTD. Because these six CTDs end with a KLIKK sequential motif, the enzymes are referred to as KLIKK proteases (Ksiazek et al., 2015b). The KLIKK proteases possess a unique structure and undergo extensive autoproteolytic processing (Cerda-Costa et al., 2011; Lopez-Pelegrin et al., 2015). Their activities, such as degrading complement proteins and LL-37 (the crucial antimicrobial peptide in the human oral cavity), may contribute to *T. forsythia* virulence through evading innate immunity (Jusko et al., 2015; Koneru et al., 2017).

In stark contrast to the other CTD proteins of *T. forsythia*, KLIKK proteases seem to be secreted directly into the extracellular medium, as shown for miropsin-2 (Tanf_06530), karilysin (Tanf_06550), and forsilysin (Tanf_06225) (Narita et al., 2014). Supporting this, proteomic analysis of the *T. forsythia* OM identified 13 of 26 proteins bearing the classical CTD, including TfsA, TfsB, and BspA (Tanf_04820), but none of the KLIKK proteases (Veith et al., 2009). Conversely, four KLIKK proteases, forsilysin, miropsin-2 (Friedrich et al., 2015), mirolase (Tanf_00440), and karilysin (Veith et al., 2015), were found in outer membrane vesicles (OMVs), although with a low Mascot score. This discrepancy could be explained by the transient presence of these proteases in the periplasm before they enter the OM translocon of T9SS. Interestingly, all three of the KLIKK proteases characterized thus far (karilysin, mirolase, and mirolysin) can remove the CTD during autoprocessing (Karim et al., 2010; Ksiazek et al., 2015a; Koneru et al., 2017). Collectively, the available data suggest that the KLIKK proteases are secreted into the extracellular milieu without removal of the CTD. This finding is similar to the secretion of PorU and PorZ from *P. gingivalis*, where the CTD is also not removed during secretion, although proteins stay associated with the cell surface (Lasica et al., 2016).

## Concluding remarks

In this review, we summarized the biochemical and structural data concerning the recently discovered T9SS identified in a majority of the bacterial species belonging to the *Bacteroidetes* phylum (Sato et al., 2010; McBride and Zhu, 2013). The system has been investigated predominantly in human oral pathogens, such as *P. gingivalis* and *T. forsythia*, and environmental saprophytes, such as *F. johnsoniae* and *C. hutchinsonii*. It seems to be a major mechanism of protein secretion in these bacteria however, some families from *Bacteroidetes* were reported to possess other secretion systems e.g., T1SS or T6SS (Russell et al., 2014; Wilson et al., 2015; Abby et al., 2016; Chatzidaki-Livanis et al., 2016; Wexler et al., 2016; Ibrahim et al., 2017). Notably, both systems allow for direct substrate translocation from bacterial cytoplasm to the cell exterior, while T9SS cargos do not omit the periplasmic space during their secretion.

The role of T9SS is to ensure cell survival and fitness in response to the microorganisms' habitat by providing transportation of proteins necessary for, among other things, virulence, nutrition, and movement (gliding motility). Hence, the variety of secreted proteins even within a single species is large and comprises numerous adhesins and hydrolytic enzymes used for attachment and degradation of large organic compounds such as proteins, cellulose, and chitin (Guo et al., 2010; McBride and Nakane, 2015).

The cargo proteins of this system (Table 2) are equipped with the classical signal peptide for Sec-dependent translocation to the IM and the conserved CTD that directs them further to the secretion machinery in the OM. The recognition signal is mostly embedded within the IgSF-like tertiary structure of the CTD (de Diego et al., 2016; Lasica et al., 2016) and likely located within the 22 amino acid residues composing the sequential motifs of PxGxYVV and KxxxK in the two most C-terminal β-strands (Shoji et al., 2011; Veith et al., 2013).

**Figure 3:**
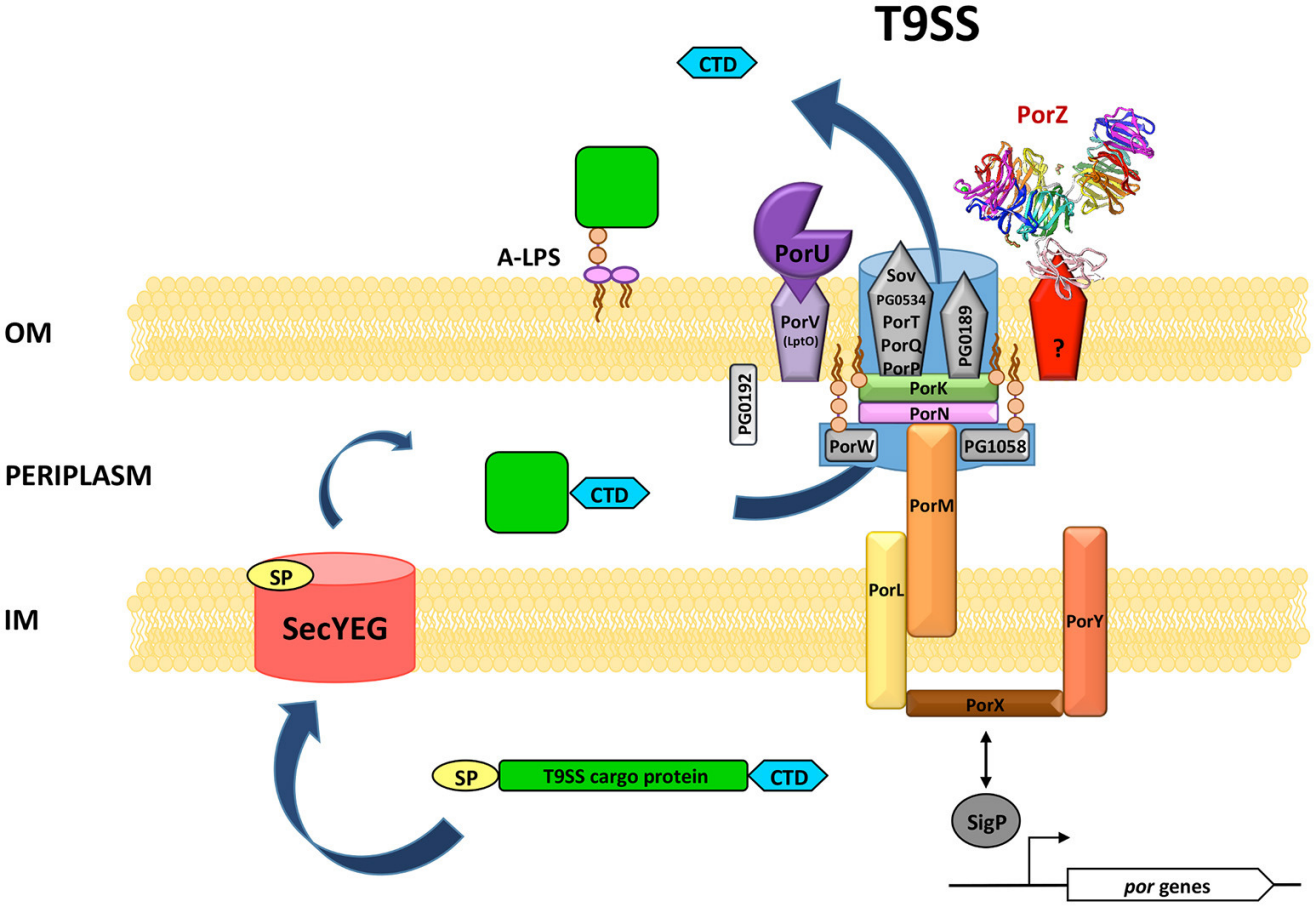
**Hypothetical model of the structure and function of ***P. gingivalis*** T9SS**. The overall translocon structure and the protein(s) forming a pore in the OM (outer membrane) have not yet been characterized. Therefore, it is shown as a background blue shape accommodating known components. Interacting proteins are situated in close proximity. OM β-barrel proteins are depicted as pentagons. PorZ is presently the only T9SS protein with the known atomic structure. The mode of its association with the translocon is not yet defined. PorK, PorW, and PG1058 are lipoproteins anchored into the inner surface of the OM. PG0192 protein precise localization and possible interactions are not known. A T9SS cargo protein is equipped with two sorting signals: N-terminal signal peptide (SP) directing the protein to the general secretion system SecYEG and conserved C-terminal domain (CTD) recognized by T9SS. After translocation through the IM (inner membrane) most proteins acquire their proper fold in the periplasm. Next, CTD directs the protein for further translocation across the OM through T9SS. Finally, CTD is cleaved off by PorU sortase and a secreted protein is modified by attachment of A-LPS resulting in the anchorage of cargo protein to the cell surface. Two component system PorX/PorY and sigma factor SigP have regulatory effect on *por* genes. Although, they are not physical elements of T9SS, PorX was shown *in vitro* to interact with PorL.

Currently, for *P. gingivalis* cells, there are 16 proteins recognized as the structural and/or functional components of the translocon and two additional elements involved in T9SS regulation (Table 1). None of these proteins are fully characterized, so their structure, mode of reciprocal interactions, and precise roles in secretion are still obscure. Nevertheless, a contemporary general concept of T9SS structure and function based on available data is presented in Figure 3. Verification of this model requires extensive structural and functional investigations to elucidate the mechanism of CTD recognition and cleavage, passage of cargos though the OM translocon, attachment of a glucan moiety, and anchoring of cargos onto the cell surface, their release into the environment, or their assembly into gliding motility machinery.

## Author contributions

AL analyzed literature, wrote the paper (excluding MK and MM sections), prepared the figures and tables. MK wrote the Mechanism of secretion and T9SS *T. forsythia* sections. MM wrote the Regulation section. JP edited the manuscript. All authors read and approved the full manuscript.

### Conflict of interest statement

The authors declare that the research was conducted in the absence of any commercial or financial relationships that could be construed as a potential conflict of interest.
